# Potentials of marine natural products against malaria, leishmaniasis, and trypanosomiasis parasites: a review of recent articles

**DOI:** 10.1186/s40249-021-00796-6

**Published:** 2021-01-22

**Authors:** Justus Amuche Nweze, Florence N. Mbaoji, Yan-Ming Li, Li-Yan Yang, Shu-Shi Huang, Vincent N. Chigor, Emmanuel A. Eze, Li-Xia Pan, Ting Zhang, Deng-Feng Yang

**Affiliations:** 1 Guangxi Key Laboratory of Marine Natural Products and Combinatorial Biosynthesis Chemistry, National Engineering Research Center of Non-Food Biorefinery, State Key Laboratory of Non-Food Biomass and Enzyme Technology, Guangxi Academy of Sciences, Nanning, 530007 Guangxi People’s Republic of China; 2 Department of Microbiology, Faculty of Biological Sciences, University of Nigeria, Nsukka, Nigeria; 3 Department of Science Laboratory Technology, Faculty of Physical Sciences, University of Nigeria, Nsukka, Nigeria; 4 College of Life Science and Technology of Guangxi University, Nanning, 530004 Guangxi People’s Republic of China; 5 Department of Pharmacology and Toxicology, Faculty of Pharmaceutical Sciences, University of Nigeria, Nsukka, Nigeria; 6 Water and Public Health Research Group, University of Nigeria, Nsukka, PMB 410001 Enugu State Nigeria; 7 National Institute of Parasitic Diseases, Chinese Center for Disease Control and Prevention, WHO Collaborating Center for Tropical Diseases, National Center for International Research on Tropical Diseases, Key Laboratory of Parasite and Vector Biology of the Chinese Ministry of Health, Shanghai, 200025 People’s Republic of China; 8 National Health Commission Key Laboratory of Echinococcosis Prevention and Control, Xizang Center for Disease Control and Prevention, Linlang North Road, Lhasa, 850000 Tibet Autonomous Region People’s Republic of China

**Keywords:** Marine natural products, Neglected tropical diseases, Anti-trypanosoma, Anti-leishmania, Anti-plasmodia, Protozoa parasites

## Abstract

**Background:**

Malaria and neglected communicable protozoa parasitic diseases, such as leishmaniasis, and trypanosomiasis, are among the otherwise called diseases for neglected communities, which are habitual in underprivileged populations in developing tropical and subtropical regions of Africa, Asia, and the Americas. Some of the currently available therapeutic drugs have some limitations such as toxicity and questionable efficacy and long treatment period, which have encouraged resistance. These have prompted many researchers to focus on finding new drugs that are safe, effective, and affordable from marine environments. The aim of this review was to show the diversity, structural scaffolds, in-vitro or in-vivo efficacy, and recent progress made in the discovery/isolation of marine natural products (MNPs) with potent bioactivity against malaria, leishmaniasis, and trypanosomiasis.

**Main text:**

We searched PubMed and Google scholar using Boolean Operators (AND, OR, and NOT) and the combination of related terms for articles on marine natural products (MNPs) discovery published only in English language from January 2016 to June 2020.

Twenty nine articles reported the isolation, identification and antiparasitic activity of the isolated compounds from marine environment. A total of 125 compounds were reported to have been isolated, out of which 45 were newly isolated compounds. These compounds were all isolated from bacteria, a fungus, sponges, algae, a bryozoan, cnidarians and soft corals. In recent years, great progress is being made on anti-malarial drug discovery from marine organisms with the isolation of these potent compounds. Comparably, some of these promising antikinetoplastid MNPs have potency better or similar to conventional drugs and could be developed as both antileishmanial and antitrypanosomal drugs. However, very few of these MNPs have a pharmaceutical destiny due to lack of the following: sustainable production of the bioactive compounds, standard efficient screening methods, knowledge of the mechanism of action, partnerships between researchers and pharmaceutical industries.

**Conclusions:**

It is crystal clear that marine organisms are a rich source of antiparasitic compounds, such as alkaloids, terpenoids, peptides, polyketides, terpene, coumarins, steroids, fatty acid derivatives, and lactones. The current and future technological innovation in natural products drug discovery will bolster the drug armamentarium for malaria and neglected tropical diseases.

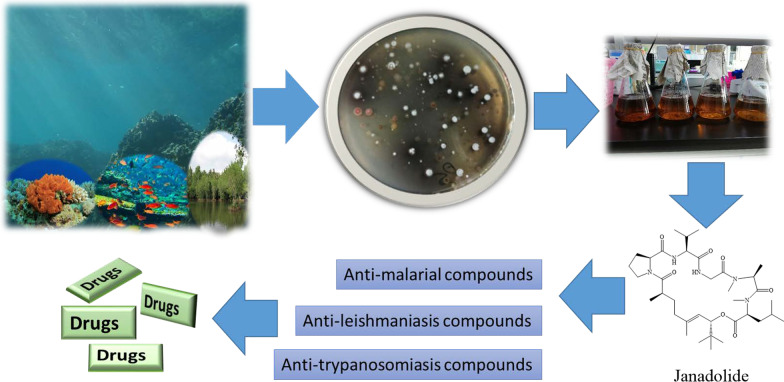

## Background

Neglected tropical diseases (NTDs) caused by protozoa such as leishmaniasis, trypanosomiasis, and malaria (no longer recognized as NTD) are the infectious diseases mainly caused by parasites, which are widespread in tropical and subtropical areas in one hundred and forty-nine countries. These diseases are the leading cause of morbidity and mortality and have affected the world’s poverty-stricken 2.7 billion people and cost emerging economies billions of dollars per year [1, 2]. Until recently, the diseases have not received as much research investment as other diseases. In the past decade, with the efforts of research institutions and some pharmaceutical companies, the situation on neglected diseases has been gradually changing. Treatments for some of these diseases take too long, are increasingly ineffective, and can be toxic, painful, and rarely accessible to infected poor people [1, 3].

Malaria, leishmaniasis, and trypanosomiasis are caused by parasitic protozoa of the genus *Plasmodium* species, *Leishmania* (over 20 *Leishmania* species), and *Trypanosoma* [*Trypanosoma brucei* complex—human African trypanosomiasis (sleeping sickness) and *Trypanosoma cruzi*—American trypanosomiasis (Chagas disease)], respectively [1, 4, 5]. According to the World Health Organization (WHO) world malaria report 2019, an estimated 228 million cases of malaria and 405 000 deaths occurred worldwide in 2018, and six sub-Saharan African countries are the most affected [1]. Leishmaniasis is widespread in tropical and subtropical regions in Latin America, Asia, and especially in Africa, and 700 000 to 1 million new cases occur annually [6]. There were fewer than 2000 cases of sleeping sickness (2017–2018) in East and West Africa, while Chagas disease affected over 8 million people in Latin America (https://www.who.int/gho/neglected_diseases/human_african_trypanosomiasis/en/). However, despite the promising nature of currently available drugs over the years, there are many limitations to their continuous usage, which suggest that introduction of new drugs is needed. These limitations range from poor absorption, resistance, lack of efficacy, toxicity and long duration of treatment. These have been discussed in details in other published review articles [7, 8].

Moreover, due to the frightening resistance of the parasites to the available drugs, the search and development of novel alternative therapeutics are imperatively necessary. Fortunately, natural products are not only very useful and active on their own but also are used as templates for the synthesis and development of other compounds [8]. Marine natural products (MNPs) are bioactive metabolites sourced from marine organisms including microbes, invertebrates and plants. MNPs from marine habitats composed of an enormous repository of diverse chemical structures and will continue to be a source of novel therapeutics, either directly in their original form or after optimization through synthetic medicinal chemistry [8, 9]. In the past few years, there has been a renaissance in natural product discovery from the marine environment. Being a unique environment, the likelihood of finding more novel bioactive molecules from this environment is enormous [10]. Some of the sources of these MNPs are bacteria, phytoplankton, sponges, red, green and brown algae, tunicates, bryozoans, soft corals, cnidarians, molluscs, plants, and echinoderms. The MNPs isolated have been classified into alkaloids, terpenoids, peptides, polyketides, steroids, fatty acid derivatives, and lactones. In addition, these MNPs have been reported to have a far-reaching biological potential like antibacterial, antifungal, anti-cancer, antiviral, anti-inflammatory, neuroprotective as well as antiparasitic activities [8, 11, 12].

It is worthy of note that in the course of this scoping review, there are other published review articles for previous years on MNPs with antiprotozoal activities [8, 12–15]. In this context, this review was aimed to show the diversity, structural scaffolds, in-vitro or in-vivo efficacy, and recent progress made in the discovery/isolation of MNPs with potent bioactivity against malaria, leishmaniasis, and trypanosomiasis parasites from marine natural products (from January 2016 to June 2020). Additionally, the challenges of MNPs antiparasitic drug discovery and possible interventions to overcome them were also discussed.

## Methods

### Study design and eligibility criteria

This scoping review was carried out following the recommendations of Preferred Reporting Items for Systematic Reviews and Meta-Analyses (PRISMA) [16].

All published articles in the English language (from 2016 to June 2020) that reported the bioactivities of marine natural products (MNPs) against the *Leishmania*, *Trypanosoma*, and *Plasmodium* parasites within the above periods were considered but only those that reported the isolation and discovery of bioactive compounds were eventually included in this scoping review.

### Search strategy

A search strategy was formed to find all scientific papers published only in English language on MNPs from 2016 up to 31st of June, 2020. PubMed and Google scholar were searched using Boolean Operators and combination of these terms: (marine natural product OR natural product OR marine NOT military) AND (leishmaniasis OR trypanosomiasis OR sleeping sickness OR Chagas’ disease OR malaria) OR (antileishmaniasis OR antitrypanosomiasis OR anti-plasmodium).

### Studies selection and data extraction

Two of the authors (JAN and FNM) separately search through the literature (PubMed and Google scholar, respectively) and the two sets of articles found were then compared. Similar and irrelevant articles were removed. The full-text of the articles were download, assessed, and the data of articles finally included were extracted.

## Results

### Study selection

**Fig. 1:**
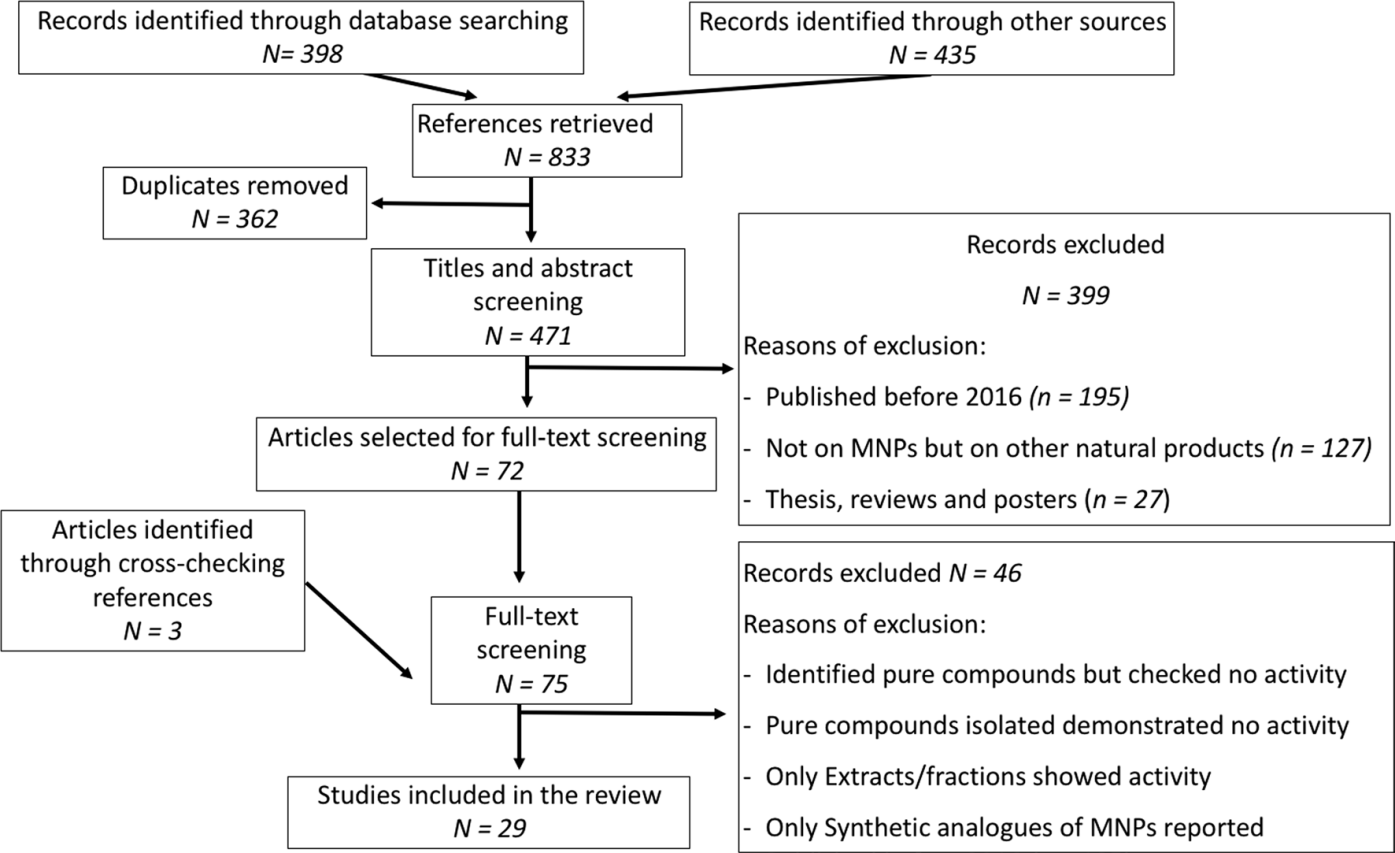
Flow of information through different phases used to identify articles included in this review

A total of 476 published articles were screened out after the removal of duplicates from a total of 836 articles downloaded independently. Full-text versions of 74 articles were retrieved after screening their titles and abstracts for further assessment. Three articles were found through cross-reference. In the end, 29 eligible articles were used in this review, as shown in Fig. 1.

### Characteristics of included studies

Studies that reported the isolation and/or identification of compounds from marine organisms with either anti-trypanosomal, anti-leishmanial or anti-plasmodial activities were considered, as summarized in Table 1. Forty five new compounds were reported to have been isolated out of a total of 125 compounds isolated. All these compounds were isolated from bacteria, a fungus, sponges, algae, a bryozoan, cnidarians and soft corals. Several potential lead MNPs with antiplasmodial activity have been reported, and in the last 5 years, few compounds have been reported to have antikinetoplastid activity against *Leishmania* and *Trypanosoma*.

**Table 1:** General summary of the articles on marine natural products and their in vitro activity included in this review

Parasite form (IC_50_ or EC_50_)	Cytotoxicity	Compounds	Classes	Sources	Organisms	Country	Refs
Marine-derived alkaloids							
*Leishmania major* (0.75 µmol/L) and *L. donovani* (7.02 µmol/L) promastigotes; *Plasmodium falciparum* 3D7 late trophozoites and schizonts (9.08 µmol/L); *Trypanosoma brucei brucei* intracellular amastigotes (0.78 µmol/L)	NT	Paenidigyamycin A (1) (Fig. 2a)	Alkaloid	Mangrove rhizosphere soil bacterium	*Paenibacillus polymyxa* strain De2sh	Ghana	[22]
*L. donovani* (115.41 µmol/L) promastigotes; *T. brucei brucei* intracellular amastigotes (28.75 µmol/L)	Mouse macrophages RAW 264.7 cells (selectivity index 8.70)	Paenidigyamycin G (2)					[23]
*L. infantum, L. amazonensis* (promastigotes)*,* and *T. cruzi* epimastigotes (NA)	HepG2 (16 ± 1 μmol/L)	Pseudoceratidine (1) and its derivatives (2–12) (Fig. 2b)	Alkaloid	Marine sponge	*Tedania braziliensis*	Brazil	[24]
*P. falciparum* 3D7 and K1 strains trophozoites and schizonts (0.96–1.24, 5.11–6.49, and 3–6 μmol/L)		Pseudoceratidine (1), 4 + 5, and 9 + 10 (inseparable structural isomers)					
*T. brucei brucei* trypanosomes (15.26 and 7.48 μmol/L)	J774.1 macrophages (IC_50_ > 200 μmol/L)	Hyrtiodoline A (1) and known compounds (2–5) (Fig. 2c)	Alkaloid	Coasts of the Red Sea sponge	*Hyrtios* sp.	Egypt	[25]
*P. falciparum* (K1 and FCR3) late trophozoites and schizonts (1.03 and 0.77 μg/ml)	MRC-5 (15.99 μg/ml)	Ceratinadin E (1)	Alkaloid (Fig. 2d)	Marine sponge	*Pseudoceratina* sp.	Japan	[26]
NA	MRC-5 (> 50 μg/ml)	Ceratinadin F (2)					
*P. falciparum* (K1 and FCR3) (3.77 and 2.45 μg/ml)	MRC-5 (12.65 μg/ml)	Psammaplysin F (3)					
*P. falciparum* 3D7 late trophozoites and schizonts (12–21 μmol/L)	HEK293 at 40 μmol/L (17–37%)	Orthoscuticellines A and B (1 and 2), orthoscuticellines C−E(3−5), and six known compounds (6−11) (Fig. 2e)	Alkaloid	Bryozoa from storm debris from Korora beach	*Orthoscuticella ventricosa*	Australia	[27]
*L. amazonensis* promastigotes and amastigote (0.06–10.65 μmol/L), *L. donovani* promastigotes (0.50– > 40 μmol/L) and *T. cruzi* epimastigote (2.86–14.56 μmol/L)	J774A.1 (8.74 ± 0.72, 5.20 ± 1.75; > 40, > 40 μmol/L)	Indolocarbazole staurosporine (STS, 1–4) (Fig. 2f)	Alkaloids	Sediment bacterium	*Streptomyces sanyensis*	Ecuador	[28]
Marine-derived terpenes and terpenoids							
*L. donovani* amastigotes (18.8 µg/ml), *T. brucei rhodesiense* trypomastigotes (11.8 µg/ml), *T. cruzi* trypomastigotes (47.8 µg/ml); and drug-resistant *P. falciparum* K1 late trophozoites and schizonts (0.65 µg/ml)	RSM L6 (56.6 μg/ml)	Bifurcatriol (1) (Fig. 3a)	Diterpene	Shore of Kilkee brown alga	*Bifurcaria bifurcate*	Ireland	[29]
*L. donovani* amastigotes (NA)	A549 (> 50 μmol/L)	Keikipukalides (1),	Diterpene (Fig. 3b)	Antarctic deep-sea octocoral—Cnidaria	*Plumarella delicatissima*	Falkland Islands (Islas Malvinas)	[30]
*L. donovani* amastigotes (1.9–12 μmol/L)		Keikipukalides (2–5), pukalide aldehyde (6), and norditerpenoid ineleganolide (7)					
*L. amazonensis* promastigotes (15.47 ± 0.26 and 36.81 ± 5.20 μmol/L) and *T. cruzi* epimastigotes ( 5.62 ± 2.48 and 35.29 ± 4.09 μmol/L)	J774.A1 (23.4 ± 5.62 μmol/L and 69.98 ± 0.14 μmol/L)	Spiralyde A (1) and 3,4-epoxy-7,18-dolabelladiene (2) (Fig. 3c)	Diterpene	Off the coast (1.5 m) brown alga	*Dictyota spiralis*	Tunisia	[31]
*L. amazonensis* and *T. cruzi* (> 100.00 μmol/L)	NT	Compounds 3–6					
*P. falciparum* strain Dd2 trophozoites (3.51, 2.11 and 0.8 µmol/L)	NT	Smenotronic acid (1), ilimaquinone (2), and pelorol (3) (Fig. 3d)	Sesquiterpenoids	Near shore sponge	*Hyrtios erectus*	Chuuk Island, Federated States of Micronesia	[32]
*L. infantum* promastigotes and amastigotes (44.9 ± 4.3 and 94.4 ± 10.1 µmol/L)	Murine macrophages (126.6 ± 21.1 and 84.5 ± 12.5 μmol/L)	(3R)- and 1(3S)-tetraprenyltoluquinol (1a/1b) and (3R)- and (3S)-tetraprenyl Toluquinone (2a/2b) (Fig. 3e)	Terpenoids	Marine macroalgae	*Cystoseira baccata*	Portugal	[33]
*L. amazonensis* intracellular amastigotes (20.2 and 22.9 μmol/L)	Mouse peritoneal macrophages (300 and 200 μmol/L)	Atomaric acid and its methyl ester derivative (Fig. 3f)	Diterpene	Brown alga snorkeling at a depth of 2–3 m	*Stypopodium zonale*	Brazil	[34]
*T. cruzi* intracellular amastigote 5.4 µmol/L)	Vero cells (21.0 ± 0.9 μg/ml)	5-chloro-1-(E)-chlorovinyl-2,4-dibromo-1,5-dimethylcyclohexane (1)	Terpenes (Fig. 3g)	Snorkeling at a depth of 2–5 m red and brown algae	*Plocamium brasiliense*	Brazil	[9]
*T. cruzi* (2 µmol/L)	Vero cells (40.2 ± 4.9 μg/ml)	Halogenated monoterpenes (F), and atomaric acid meroditerpene (2)			*Stypopodium zonale*		
Promastigotes of *L. amazonensis, L. donovani* and epimastigote of *T. cruzi* (5.40–46.45 μmol/L)	J774A.1 (> 100 µmol/L)	Oxasqualenoid metabolites (1–11) (Fig. 3h)	Polyether triterpenoids	Marine red alga	*Laurencia viridis*	Spain	[35]
*T. cruzi* trypomastigote (32 μmol/L) and intracellular amastigotes (40 μmol/L)	BMM (> 200 μmol/L)	Isololiolide (Fig. 3i)	Carotenoid	Cnidarian got at depth of 1 to 14 m	*Macrorhynchia philippina*	Brazil	[36]
*P. falciparum* 3D7 (80 µmol/L)	Jurkat, MDA-MB-231, U2OS, and A549 cell lines (24.9, 32.3, 41.7 and > 100 μmol/L)	Sinuketal (1),	Terpenoids (Fig. 3j)	South China Sea soft corals	*Sinularia* sp.	Yongxing Island, China	[37]
*P. falciparum* 3D7 (NT)	HeLa, HCT-116 and 11.6, 33.3 and > 100 μmol/L)	Sinulins A and B (2 and 3), sinulins C and D (4 and 5), sesquiterpenoids (6–13) and cembranoids (14–21)					
Marine-derived amino acids, peptide, amides, and polyketide							
*T. brucei brucei* trypanosomes (47 nmol/L)	MRC-5 cells (> 10 μmol/L)	Janadolide (Fig. 4a)	Cyclic polyketide-peptide	Marine coast cyanobacterium	*Okeania* sp.	Japan	[38]
*P. falciparium* late trophozoites and schizonts (0.14 μmol/L)	MRC-5 cells (> 10 μmol/L)	Ikoamide (Fig. 4b)	Lipopeptide		*Okeania* sp.	Japan	[39]
*P. falciparum* late trophozoites and schizonts (0.52 and 1.0 μmol/L)	HeLa cells (10 μmol/L)	Hoshinoamides A (1) and B (2) (Fig. 4c)	Lipopeptides		*Caldora penicillata*	Japan	[40]
*T. brucei* brucei GUT trypanosomes (IC_50_ = 6.1 nmol/L)	MRC-5 cells (IC_50_ > 25 μmol/L)	Hoshinolactam (1) (Fig. 4d)	Lactam		*Oscillatoria* sp.	Japan	[41]
*P. falciparum* 3D7 and Dd2 ring stage (777.9–598.5 nmol/L)	HEK-293 cells (Only comp. 2–cytotoxic)	Herbimycin G (1) and elaiophylin (2), Cyclo-l-Pro-l-Leu (3), Cyclo-l-Pro-l-Phe (4), Cyclo-l-Pro-l-Val (5), Cyclo-l-Pro-l-Tyr (6) (Fig. 4e)	Polyketides	Bacterium from ascidian *Symplegma rubra*	*Streptomyces* sp. (USC-16018)	Australia	[42]
*P. falciparum* blood-stages (0.99 and 1.5 µmol/L)	HEK293T (> 4.8 and 19 μmol/L), HepG2 (NT and > 23 μmol/L)	Ulongamide A (2), lyngbyabellin A (3),	Peptide	Reef slopes offshore cyanobacterium	*Moorea producens*	Island in Fiji	[43]
Liver-stage *P. berghei* liver schizonts (EC_50_ = 11, 7.1, and 4.5 µmol/L)	HEK293T (> 23, > 31, and > 13 μmol/L), HepG2 (> 23, 17, and > 13 μmol/L)	Kakeromamide B (1), 18E-lyngbyaloside C (4), and lyngbyaloside (5) (Fig. 4f)					
Marine-derived quinones, macrolide, lactones, and sterol							
*T. brucei* strain TC221 trypanosomes (3.38 (48 h) and 5.26 μmol/L (72 h)	Macrophages (J774.1) (> 200 μmol/L)	Fridamycin H (1)	Anthraquinones (Fig. 5a)	Bacterium from red Sea sponge	*Actinokineospora spheciospongiae* sp. nov	Egypt	[44]
NA		Fridamycin I (2), actinosporin C (3), D (4), and G (5)					
*P. falciparum* Dd2 in blood-stage (223 nmol/L) and intracellular *L. donovani* (4.67 μmol/L)	HepG2 (extract not cytotoxic 25 μg/ml)	Palstimolide A (Fig. 5b)	Macrolide	Central Pacific Ocean cyanobacterium	*Leptolyngbya* sp.	USA	[45]
*P. falciparum* strain HB3 (NA and 5.7 ± 0.7 μmol/L)	NT	Bastimolide A (1) and B (2) (Fig. 5c)	Macrolide	Tropical marine cyanobacterium	*Okeania hirsute*	USA	[46]
Active against stages/forms of *P. falciparum; L. infantum* amastigote (7.64 and 3.19 µmol/L) and promastigotes (28.1 and 7.42 µmol/L), and *L. tropica* promastigotes (20.28 and 7.08 µmol/L)	HMEC‐1 (62.19 ± 1.98 and 36.85 ± 5.79 µmol/L); THP‐1 (> 100 and 31.75 µmol/L)	Sesquiterpene avarone (1) and avarol (3) (Fig. 5d)	Quinone	Coast area sponge	*Dysidea avara*	Turkey	[47]
*L. amazonensis* promastigotes forms (5.25 µg/ml) and intracellular amastigotes (18.18 µg/ml)	NT	Harzialactone A (Fig. 5e)	Lactone	Marine fungus	*Paecilomyces* sp. 7A22	Brazil	[48]
chloroquine-resistant *P. falciparum* 3D7 strain blood stage (3.0 μmol/L and NA)	RAW 264.7 cells and N2A cells (Not cytotoxic)	Halymeniaol (1) and cholesterol (2) (Fig. 5f)	Sterol	Arabian sea red alga	*Halymenia floresii*	India	[49]
*P. falciparum* 3D7 strains blood stage (359 and 0.250 nmol/L)	NT	Kaimanol (1) and saringosterol (2)	Sterol	Marine marine sponge	*Xestospongia* sp.	Indonesia	[50]

*NT* not tested, *NA* not active, *HepG2* human hepatoma cell line, *HEK293* human embryonic kidney cell line, *J774A.1* macrophage cell line, *RSM L6* rat skeletal myoblast L6 cells, *A549* human lung carcinoma cells, *J774.A1* murine macrophage cell line (ATCC # TIB-67), *BMM* bone-marrow derivate macrophages, *MRC-5* human fetal lung fibroblast, *HeLa cells* human epithelial cells, *HMEC‐1* human microvascular endothelial cell line, *THP‐1* human leukemic cell line

### Marine natural products drug discovery and their potentials against the three NTDs and malaria

Over the past decade, some bioactive MNPs have been isolated, characterized and studied extensively particularly from the vast domain of marine organisms, for example, brown and red algae, and cyanobacteria. Interestingly, there are thousands of reports on the antibiotic or antiparasitic activity of extracts, fractions, and pure compounds from marine organisms.

Nonetheless, the majority of these studies did not proceed to extensively evaluate the isolated metabolites for further development and application. That could explain why not even a single marine-derived antiparasitic substance has been put to use or supersedes compounds from other natural products (plant and terrestrial organisms), purely synthetic, and semi-synthetic products [17]. At the end of 2018, more than 28 600 MNPs were identified, and the majority of their biological activities were based on cytotoxic and anticancer properties [18]. However, considering the ecological role of MNPs as a chemical defence in organisms, it was not a surprise. The source of research funding for MNP drug discovery may have contributed to the greater emphasis given to antitumor activity, according to some researchers [19].

Nevertheless, the earnest efforts contributed all over the world by researchers with a focus on MNPs discovery have been able to evolve a few potential antiparasitic compounds from more than a few marine organisms, namely the cyanobacterial linear lipodepsipeptide, symplostatin 4 (Sym4)/gallinamide A containing a methylmethoxypyrrolinone (MMP) moiety from *Schizothrix* sp., and carmaphycin (two leucine-derived a,b-epoxyketone containing modified peptides) from another marine cyanobacterium *Symploc*a sp. [20] (studied extensively) [21]. Other isolated marine natural products need to follow suit. These compounds have shown that marine-world evidently and explicitly has enormous novel lead molecules required in the development of potent therapeutic agents that are active against a variety of parasites. Consequently, with the recent developmental strides and introduction of advanced computer-aided sophisticated analytical instruments, the dream of novel antiparasitic drugs from MNPs will soon be turned like fiction into reality. Recent structural scaffolds, in-vitro efficacy and some other pharmacological properties were outline in Table 1.

#### Marine-derived alkaloids

**Fig. 2:**
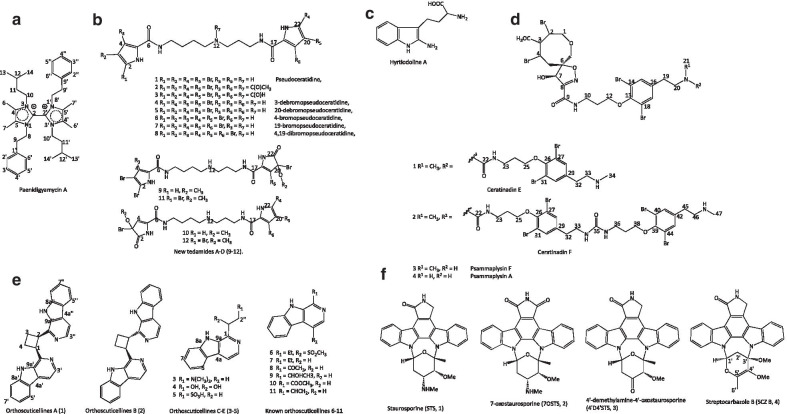
Structures of some of the marine derived-alkaloids

Antiparasitic imidazole alkaloid, paenidigyamycin A (1) (Fig. 2a) produced by Ghanaian *Paenibacillus polymyxa* strain De2sh inhibited *L. major* (IC_50_ = 0.75 µmol/L) as effective as the conventional antibiotic, amphotericin B (IC_50_ = 0.31 µmol/L), but 20 folds less active against *L. donovani* (IC_50_ = 7.02 µmol/L). The same imidazole alkaloid was as well ten-fold more effective against *T. brucei brucei* (IC_50_ = 0.78 µmol/L) than laboratory standard *Coptis japonica* (IC_50_ = 8.20 µmol/L). Interestingly, the compound also inhibited *P. falciparum* 3D7 but was not as strong as artesunate (IC_50_ = 36 nmol/L) [22]. Paenidigyamycin G (2), a derivative of compound (1), showed moderate to weak antiparasitic activity against *T. brucei brucei* and *L. donovani* cells (IC_50_ = 115.41 and 28.75 µmol/L), though, its toxicity profile on *T. brucei brucei* in the presence of mouse macrophages RAW264.7 cell lines was relatively low with a selectivity index (SI) of 8.70 [23].

A series of bromopyrrole alkaloids isolated from a marine sponge, *Tedania braziliensis* (Rio de Janeiro state) was made up of new pseudoceratidine (1) and its derivatives (2–12) as shown in Fig. 2b. The compounds exhibited no activity against promastigotes of *L. infantum* and *L. amazonensis,* and *T. cruzi* epimastigotes, but were (compounds 1, 4 + 5, 9 + 10) potent against sensitive (3D7 strain) and resistant (K1 strain) *P.* *falciparum* strains (0.96–1.24, 5.11–6.49, and 3–6 μmol/L) (Table 1) [24].

Compounds 4 (3-debromopseudoceratidine) and 5 (20-debromopseudoceratidine) were inseparable mixtures with one more hydrogen atom and one fewer bromine atom than compound (1). The cytotoxicity study using human liver cancer HepG2 cell line showed that compound (1) was the most toxic (16 ± 1 μmol/L) (SI 1.5), and other compounds 16, 4, 5, 23, 25, 31 and 50 had generally weak cytotoxicity (SI: 35–125). SAR studies of pseudoceratidine and 23 of its synthetically produced derivatives, showed that the expression of antiplasmodial activity by both the natural and seven synthetic pseudoceratidine may be due to the polyamine chain length bearing basic nitrogen and bromine atoms attached on furan or pyrrole terminal moieties. For instance, good antiplasmodial activity was observed with *N*-Methylpseudoceratidine (16) (4 ± 1 μmol/L), compound (23) (a large polyamine chain bearing two basic nitrogens) (3 ± 1 μmol/L), and furan derivative (50) (bearing four bromine atoms) (3 ± 1 μmol/L), while a twofold decrease in activity value was found in compound (25) (2 ± 1 μmol/L). Compound (31), 2,21-debromopseudoceratidine, had weaker antiplasmodial activity (7 ± 1 μmol/L) relative to compound 1 (1) [24]. This study has projected pseudoceratidine as a propitious scaffold for new antimalarial drugs development.

The metabolomics analysis of a crude extract of the sponge, *Hyrtios* sp. with antitrypanosomal activity, resulted in the isolation of a new alkaloid, hyrtiodoline A (1) (Fig. 2c) and other already known compounds (2–5). The compounds were not cytotoxic against J774.1 macrophages (IC_50_ > 200 μmol/L), although most of them exhibited no inhibitory activity against *T. brucei brucei*, but compound (1) showed activity after 48 h (IC_50_ = 15.26 μmol/L) and 72 h (IC_50_ = 7.48 μmol/L) [25].

Two strains of *P. falciparum* (drug-resistant K1 and drug-sensitive FCR3) were inhibited by bromotyrosine alkaloid, ceratinadin E (1) (1.03 and 0.77 μg/ml respectively) produced by Okinawan marine sponge (*Pseudoceratina* sp.), alongside other compounds (Fig. 2d), ceratinadin F (2) (no activity) and psammaplysin F (3) (3.77 and 2.45 μg/ml respectively), with weak cytotoxicity against MRC-5 (15.99, > 50, and 12.65 μg/ml). However, chloroquine (3 and 22, 11 and 70 fold) and artmisinin (103 and 87.5, 377 and 278 fold) had higher activity than the isolated compounds [26].

From an Australian bryozoan, *Orthoscuticella ventricosa,* two new orthoscuticellines A and B (1 and 2), three new β-carboline alkaloids, orthoscuticellines C−E (3−5), and six other known compounds (6–11) (Fig. 2e), were isolated. The two bis-β-carbolines (orthoscuticellines A and B) has a cyclobutane moiety. The compounds showed moderate activity against the same parasite (12–21 μmol/L) while some had cytotoxicity effects on human embryonic kidney cells (40 µmol/L) [27].

Antikinetoplastid natural indolocarbazole staurosporine (STS, 1–4) (Fig. 2f) isolated from *Streptomyces sanyensis* were compared with similar and commercially available compounds: rebeccamycin (5), K252a (6), K252b (7), K252c (8), and arcyriaflavin A (9) against *L. amazonensis, L. donovani and T. cruzi*. Compound (2), 7-oxostaurosporine was the most active (IC_50_ = 3.58 ± 1.10, 0.56 ± 0.06 and 1.58 ± 0.52 μmol/L, respectively) against the parasites similar to that of compound STS (1), and as well had a selectivity index (IC_50_) of 52 against the *L. amazonensis* amastigote compared to the Mus musculus ascites reticulum cell (J774A.1). The researchers went further to show that since the two compounds have similar structural features, orientation and conformation (lactam group and the methyl amine at their C-4′ position), they interact with the conserved aminoacidic residues in protein kinases of the parasite, which is essential for the parasite survival. More studies are needed to further develop these compounds as potential antiparasitic drugs [28].

#### Marine-derived terpenes and terpenoids

**Fig. 3:**
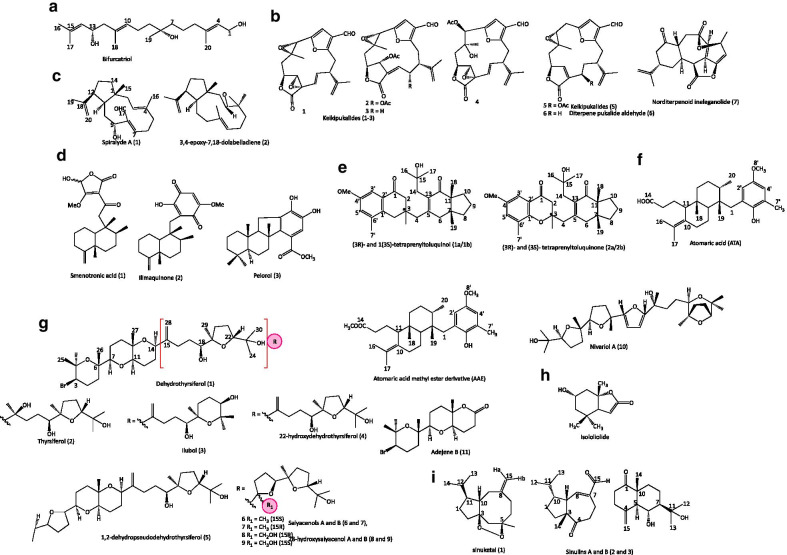
Structures of some of the isolated terpenes and terpenoids

A new linear diterpene, bifurcatriol (1) (Fig. 3a) was isolated with previously reported compounds, elegandiol (2) and bifurcane (3), from an Irish brown alga, *Bifurcaria bifurcate.* The compound (1) exhibited moderate in vitro antiprotozoal activity against *L. donovani* (18.8 µg/ml), *T. brucei rhodesiense* (11.8 µg/ml), and *T. cruzi* (47.8 µg/ml); and inhibited drug-resistant *P. falciparum* K1 at low concentration (0.65 µg/ml) and was moderately cytotoxic to RSM L6 cell lines (56.6 μg/ml). Bifurcatriol (1) is the first reported acyclic diterpene with two stereogenic centres. It has potential as a lead compound whose antiplasmodial activity can be enhanced by medicinal chemistry approaches [29].

An Antarctic deep-sea Cnidaria (order Alcyonacea), octocoral *Plumarella delicatissima* from Falkland Islands (Plateau of Fascination) yielded five furanocembranoid diterpenes, keikipukalides (1–5) and diterpene pukalide aldehyde (6), and norditerpenoid ineleganolide (7) (Fig. 3b). This family of diterpenes (2–7) exhibited antileishmanial activity against *L. donovani* (IC_50_ = 1.9–12 μmol/L) and showed no cytotoxic effect against human lung carcinoma cells A549 (> 50 μmol/L) [30].

Bioassay-activity-guided fractionation of an extract with antikinetoplastidal activity from brown alga *Dictyota spiralis* (Northwest of Tunisia) has resulted in the isolation and identification of a new dolabellane aldehyde, spiralyde A (1), and other known diterpenes (2–6). Only Spiralyde A (1) and 3,4-epoxy-7,18-dolabelladiene (2) (Fig. 3c) exhibited moderate inhibitory activity against *L. amazonensis* (IC_50_ = 15.47 ± 0.26 and 36.81 ± 5.20 μmol/L) and *T. cruzi* (5.62 ± 2.48 and 35.29 ± 4.09 μmol/L), and cytotoxic activity against J774.A1 cells (23.4 ± 5.62 μmol/L and 69.98 ± 0.14 μmol/L)*.* The spiralyde A (**1**) is the most potent antitrypanocidal dolabellane akin to benznidazole, the reference drug [31].

Three potent antimalarial sesquiterpenoids, smenotronic acid (1), ilimaquinone (2), and pelorol (3) (Fig. 3d), isolated from sponge *Hyrtios erectus* showed promising in vitro activity against chloroquine-resistant *P. falciparum* strain Dd2 at IC_50_ values of 3.51, 2.11 and 0.8 µmol/L, respectively [32]. The same sesquiterpene derivatives have been isolated from sponges, and ilimaquinone (2) was shown to inhibit nitric oxide (NO) production in lipopolysaccharide-stimulated in BV2 microglia cells (IC_50_ = 10.40 ± 1.28 µmol/L) [51].

Two meroditerpenoids and two tetraprenyltoluquinone (Fig. 3e) isolated and identified from hexane extract of macroalgae, *Cystoseira baccata* had antileishmanial activity against *L. infantum* promastigotes (IC_50_ = 44.9 ± 4.3 and 94.4 ± 10.1 µmol/L, respectively) and low cytotoxicity against murine macrophages (126.6 ± 21.1 and 84.5 ± 12.5 μmol/L) [33]. The first compound has more prospect for further studies and development for canine and human visceral leishmaniases. Similarly, a lipophilic extract of brown alga *Stypopodium zonale* led to the isolation of anti-*Leishmania* meroditerpene compounds, atomaric acid, and its methyl ester derivative (Fig. 3f). The compounds had moderate leishmanicidal activity against intracellular amastigotes of *L. amazonensis* (IC_50_ = 20.2 and 22.9 μmol/L) as well as low cytotoxicity against mouse peritoneal macrophages (300 and 200 μmol/L) with selectivity indexes of 8.4 and 11.5 μmol/L [34]. Also, the atomaric acid showed enhanced trypanocidal activity with low selectivity against *T. cruzi* (amastigotes and trypomastigotes) (IC_50_ < 10 µmol/L) [9].

Marine polyether triterpenoids isolated from a seasonal and different red alga *Laurencia viridis* (Paraiso Floral, Tenerife, Canary Islands) were elucidated to be 16 oxasqualenoid metabolites (1–11) as shown in Fig. 3g. Eight of these compounds showed potent antikinetoplastid against *L. amazonensis*, *L. donovani* and *T. cruzi* activities (IC_50_ = 5.40–46.45 μmol/L). Interestingly, 28-iodosaiyacenol B (15) which was produced through semi-synthetic means inhibited *L. amazonensis* (IC_50_ = 5.40 μmol/L) and was not cytotoxic to J774A.1 cells (IC_50_ > 100 μmol/L). The compound is a good candidate for a new antikinetoplastid drugs development since its activity was comparable with the reference compound, miltefosine (IC_50_ = 6.48, 72.19 μmol/L) [35].

A carotenoid metabolite, isololiolide (Fig. 3h) with antikinetoplastid potential isolated through bio-guided fractionation of culture broth of cnidarian, *Macrorhynchia philippina* (Sao Paulo State, Brazil), exhibited in vitro activity against both trypomastigote (IC_50_ = 32 μmol/L) and intracellular amastigotes (IC_50_ = 40 μmol/L) of *T. cruzi.* Isololiolide is a promising drug that needs further investigation since it presented no cytotoxicity against mammalian bone-marrow derivate macrophages (BMM) (> 200 µmol/L) [36].

Qin et al. in a study which showed that soft corals are rich in natural products exploited *Sinularia* sp., and isolated 21 terpenoids: sinuketal (1), sinulins A and B (2 and 3) (sesquiterpenoids), sinulins C and D (4 and 5) (cembranoids) (Fig. 3i), known sesquiterpenoids (6–13) and cembranoids (14–21). Unexpectedly, the first marine-derived compound having isopropyl-type bicyclo [6.3.0] undecane carbon skeleton with unique endoperoxide moiety is sinuketal (1). Compared with its analogue, tehranolide, the positions of the isopropyl and methyl groups were quite different. In in vitro antimalarial activity against *P. falciparum* 3D7, endoperoxide, sinuketal (1) showed mild activity (IC_50_ = 80 µmol/L) as well as weak cytotoxicity against Jurkat, MDA-MB-231 (24.9 μmol/L), U2OS (32.3 μmol/L), and A549 (> 50 μmol/L) cell lines, and mild inhibitory acetylcholinesterase activity [37].

#### Marine-derived amino acids, peptide and polyketide

**Fig. 4:**
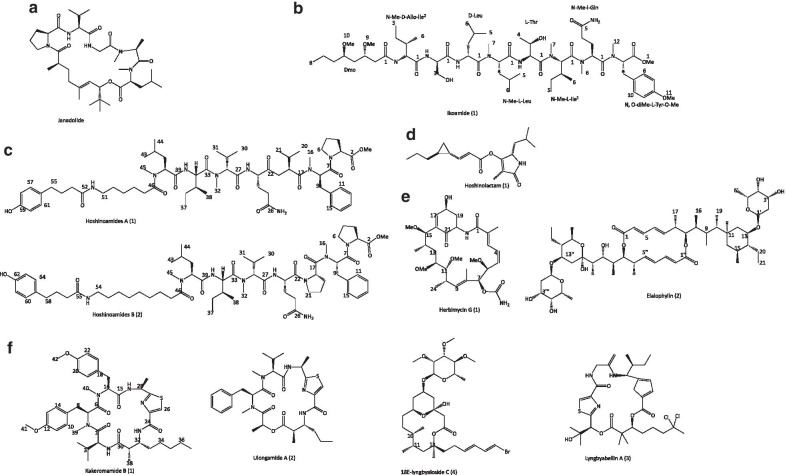
Structures of some of the isolated amino acids, peptide, amide and polyketide

Janadolide is a new cyclic polyketide-peptide hybrid (23-membered macrocyclic depsipetide) (Fig. 4a) possessing a tert-butyl group isolated from a cyanobacterium, *Okeania* sp. near Janado, Okinawa. The compound exhibited potent activity against *T. brucei brucei* (IC_50_ = 47 nmol/L) which was stronger than the commonly used therapeutic drug, suramin (IC_50_ = 1.2 µmol/L), and had no cytotoxic effect against MRC-5 cells (> 10 μmol/L) [38]. Further biological evaluation of synthetically produced des-tert-butyl janadolide, suggested that the tert-butyl group is necessary for its antitrypanosomal activity [52]. Recently there is another report on lipopeptide with strong and selective antimalarial activity isolated from the same cyanobacterium called ikoamide (Fig. 4b). The compound inhibited the late stage of trophozoites and schizonts of *Plasmodium* parasite (IC_50_ = 0.14 μmol/L) without cytotoxicity against MRC-5 cells (> 10 μmol/L). More studies on these compounds will provide new insight for its development as a new drug for the treatment of malaria [39]. Previously, Iwasaki and his group isolated another two linear lipopeptides from cyanobacterium in a new genus named *Caldora penicillata,* with a hydroxyphenylbutanoic acid moiety and an unusual long-chain amino acid moiety. The compounds, hoshinoamides A (1) and B (2) (Fig. 4c) had moderate inhibitory activity against *P. falciparum* at IC_50_ value of 0.52 and 1.0 μmol/L, respectively, and as well had no cytotoxicity effect on HeLa cells (> 10 μmol/L) [40]. The same group also reported the isolation and screen of a potent antitrypanosomal compound, hoshinolactam (1) (Fig. 4d) from *Oscillatoria* sp. and its synthetic analogue, which like the reference drug, pentamidine (IC_50_ = 4.7 nmol/L), strongly inhibited trypanosomes of *T. brucei brucei* GUT (IC_50_ = 6.1 and 3.9 nmol/L) and unlike the reference drug (16.8 μmol/L) had no cytotoxicity against MRC-5 cells (IC_50_ > 25 μmol/L) [41].

A bacterium, *Streptomyces* sp. (USC-16018) associated with an Australia ascidian *Symplegma rubra* yielded ansamycin polyketide (1), macrocyclic polyketide (2) as shown in Fig. 4d, and other four known diketopiperazines (3–6). The two polyketides (1–2) demonstrated inhibitory activity (> 75%) against strains of *P. falciparum* (3D7 and Dd2) (777.9–598.5 nmol/L) at ring stages. Although, compound 2 was highly cytotoxic (HEK-293 cells—IC_50_ = 1445 nmol/L); compound 1 was not and was water-soluble as expected [42].

Sweeney-Jones et al. isolated a new cyclic peptide, kakeromamide B (1), along with other compounds ulongamide A (2), lyngbyabellin A (3), 18*E*-lyngbyaloside C (4) (Fig. 4e), and lyngbyaloside (5) from the marine cyanobacterium *Moorea producens* got from reef slopes offshore of Tuvuca Island in Fiji. Compounds 1, 2 and 3 exhibited anti-parasitic activity against *P. falciparum* blood-stages (EC_50_ = 8.9, 0.99 and 1.5 µmol/L, respectively) while compounds 1, 4, and 5 showed moderate activity against liver-stage *P. berghei* liver schizonts (EC_50_ = 11, 7.1, and 4.5 µmol/L, respectively). The compounds had no cytotoxic effect on HEK293T (> 23, > 31, and > 13 μmol/L) and HepG2 (> 23, 17, and > 13 μmol/L) cell lines. It was postulated that kakeromamide B interferes with the host cells invasion by the parasite through its interaction with actin-like proteins and a sortilin protein in the parasite [43].

#### Marine-derived quinones, macrolide, lactones, and sterol

**Fig. 5:**
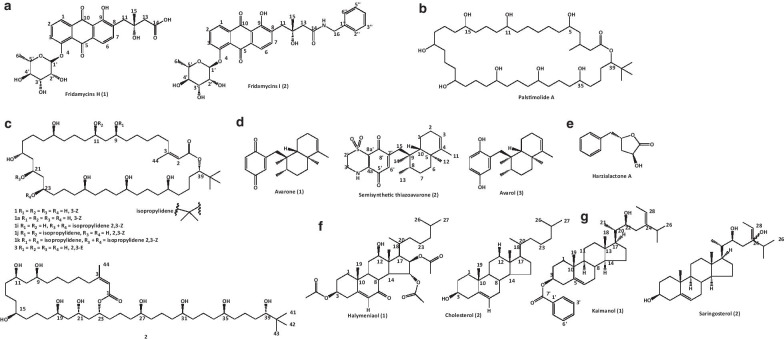
Structures of some isolated quinones, macrolide, lactones, and sterol

An actinomycete, *Actinokineospora spheciospongiae* sp. nov. associated with a red sponge (*Spheciospongia vagabunda*) (Ras Mohamed, Egypt) was primed with *N*-acetylglucosamine in solid culture. This led to the isolation of two new anthraquinones, fridamycins H (1) and I (2) (Fig. 5a), and other known compounds actinosporin C (3), D (4), and G (5). Fridamycin H showed significant antitrypanosomal activity against *T. brucei* strain TC221 at IC_50_ values of 3.38 and 5.26 μmol/L, respectively after 48 and 72 h, and had no cytotoxic activity against macrophages (J774.1) (> 200 μmol/L). It is a strong indication or evidence that the strategy is effective and has the potential to trigger expression of natural products [44].

A macrolide with a 40-membered ring, palstimolide A (Fig. 5b), isolated from Central Pacific Ocean Cyanobacterium exhibited interesting anti-parasitic activities against blood-stage of the *P. falciparum* Dd2 (IC_50_ = 223 nmol/L) and intracellular *L. donovani* (IC_50_ = 4.67 μmol/L), as well as low toxicity [45]. Likewise, another new 24-membered polyhydroxy macrolide bastimolide B (2) was isolated along with a known bastimolide A (1) (Fig. 5c) from a cyanobacterium, *Okeania hirsute*. The new compound (2) has unique terminal tertbutyl group and a long aliphatic chain, and the importance of double bond, the 1,3-diol and 1,3,5-triol functionalities in the six analogues of the compound, was revealed in their structure−activity relationship studies using based on its six analogues. Compound (1) exhibited strong anti-plasmodial activity against chloroquine-sensitive *P. falciparum* strain HB3 (IC_50_ = 5.7 ± 0.7 μmol/L). [46].

Interestingly, the antiparasitic properties of a sesquiterpene avarone (1), its reduced form avarol (3), isolated from sponge *Dysidea avara* and thiazoavarone (2) (semisynthetic thiazinoquinone) (Fig. 5d), were pharmacologically investigated. The three compounds especially avarol (3) were very active against different stages of *P. falciparum* asexual and sexual forms (gametocytes stage V) (IC_50_ = 15.53 ± 5.26, 15.01 ± 3.19 and 9.30 ± 1.90 µmol/L), while compound (2) was the most active against chloroquine‐sensitive (CQ‐S) D10 (2.74 ± 0.51, 0.38 ± 0.15, and 0.96 ± 0.24 µmol/L) and a chloroquine‐resistant (CQ‐R) W2 (2.09 ± 0.52, 0.21 ± 0.03, and 1.10 ± 0.15 µmol/L) strains. The study showed that most of the compounds had a cytotoxic effect on microvascular endothelial (HMEC‐1) (62.19 ± 1.98, 3.31 ± 1.53, and 36.85 ± 5.79 µmol/L) and acute monocytic leukemia (THP‐1) (> 100, 7.41, and 31.75 µmol/L) cells. Also, the *L. infantum* promastigotes (7.42–28.21 µmol/L) and amastigotes (7.64, 4.99, and 3.19 µmol/L),* L. tropica* promastigotes (7.08–20.28 µmol/L), and *S. mansoni* schistosomula (5.90–42.77 µmol/L) were all inhibited. The 1,1‐dioxo‐1,4‐thiazine ring may have favoured higher activity in semisynthetic thiazinoquinone than other compounds [47].

An antileishmanial compound, harzialactone A (Fig. 5e), isolated from a fungus, *Paecilomyces* sp. 7A22 showed potent activity against promastigotes forms (IC_50_ = 5.25 µg/ml) and intracellular amastigotes (IC_50_ = 18.18 µg/ml) of *L. amazonensis*, though, less potent than reference antibiotic, amphotericin B (0.119 and 0.095 µg/ml) [48].

A new antimalarial mono-hydroxy acetylated sterol derivative, halymeniaol (1), along with a known compound, cholesterol (2) (Fig. 5f) were isolated from marine alga *Halymenia floresii.* The former exhibited inhibitory activity against chloroquine-resistant *P. falciparum* 3D7 strain (IC_50_ = 3.0 μmol/L) and was not cytotoxic to RAW 264.7 and N2A cells [49]. Sterols, kaimanol (1) and saringosterol (2) (Fig. 5g), with significant antimalarial activity against *P. falciparum* 3D7 strains (IC_50_ = 359 and 0.250 nmol/L respectively) was isolated from an Indonesian marine sponge, *Xestospongia* sp. *n*-hexane extract. This study went further to postulate that the antiplasmodial activity of a sterol structure is good in the presence of an olefinic moiety and reduced due to the presence of benzoyl moiety [50].

## Discussion

In developing countries, NTDs and malaria remain a public health issue, which may well have been underrated, as pointed out earlier in this review. Notwithstanding, these ancient diseases have continued to afflict a large population of people in poor communities around the world. Besides the increase in the substantial amount of funding, high-quality research, and drug development for malaria, other NTDs are yet to receive the required attention in terms of investment and the development of chemotherapeutic interventions, relatively due to the likelihood of poor financial returns on investment [2].

The search for novel bioactive compounds, to combat malaria and especially NTDs from marine organisms, has yielded some good results with the isolation of compounds with confirmed IC_50_ values of less than one micro-molar, no or low cytotoxicity and high selectivity index. The activities of these isolated compounds were screened using in vitro cell-based techniques and live parasites, which is a starting point and a step ahead of target-based screen technique in the drug discovery pipeline, and since it is already known to kill the parasite, the cellular permeability issue has been addressed.

In terms of antileishmanial and antitrypanosomal activities, some of reported MNPs previously mentioned, for instance, imidazole alkaloid, paenidigyamycin A (1) [22] and its paenidigyamycin G (2) [23] were found to consistently exert potent leishmanicidal activity, strong antitrypanosomal activity, relatively low cytotoxicity, as well as antibacterial activity. The broad spectrum of activities of paenidigyamycin is due to the presence of a special structural features and electron-rich environment, that is, imidazole moiety. The nitrogen-containing heterocycles are important and are widely explored and utilized for drug discovery by the pharmaceutical industry because of their physicochemical properties which makes them ideally suited for binding (weak or strong) to a variety of therapeutic targets. Indolocarbazole staurosporines and alkaloids (STS, 1–4) were active against the parasites; less toxic to murine macrophage J774A.1 than the reference compounds, miltefosine and benznidazole, and compound 2 had selectivity index twofold the value got for miltefosine, the reference drug for the treatment of leishmaniasis. Based on the structure–activity relationship study, the likely mechanism of action of STS 1–4 was suggested to be the inhibition of parasite protein kinases, and the sugar moiety in the compounds was confirmed to be relevant in the protein kinases inhibition [28].

Intriguingly, janadolide is another reported cyclic polyketide−peptide with potent antitrypanosomal activity and no cytotoxicity against human cells [38]. The compound has been totally synthesized through lithiation of vinyl iodide, its addition to a Weinreb amide with a *tert*-butyl group and stereoselective 1,2-reduction, and finally macrolactamization [53]. Recently, other researchers synthesized the same compound and small library (18a–h) of its simplified analogues via an enantioselective (−)-B-chlorodiisopinocampheylborane-mediated reduction and a B-alkyl Suzuki reaction. Surprisingly, janadolide and its eight analogues had antitrypanosomal activity against pathogenic *T. brucei* rhodesiense and *T. cruzi* (IC_50_ = 47 nmol/L) parasites but were inactive against *L. donovani.* Unlike the clinically approved reference drug, melarsoprol, the compounds were not cytotoxic to human L6 cell lines at high concentration (100−150 μmol/L). A relatively flat (IC_50_ = 33−104 μmol/L) structure−activity data was generated, suggesting that the activity of the compounds will not be compromised when the amide bonds are replaced with ester linkage and olefin moiety [54]. The discovery and development of MNPs as potential antimalarial medicines is still in an infant stage, although it has progressed far more than other MNPs with antiparasitic activity. These compounds, ikoamide [39], hoshinoamides A and B (1–2) [40], ulongamide A (2) and lyngbyabellin A (3) [43] isolated from marine cyanobacteria, at low concentration especially ikoamide, selectively inhibited blood-stage of *P. falciparum* without cytotoxicity to cell lines used. Similar to pentamidine, hoshinolactam and especially its synthetic analogue showed antitrypanosomal activity but unlike the clinical used drug, there was no cytotoxicity against MRC-5 cells, as explained previously (IC_50_ > 25 μmol/L) [41].

Some of the important factors that need researchers’ attention in MNPs antiparasitic drug discovery are novel chemical motifs, the absence of host cell cytotoxicity, sub-micromolar potency, mode of action, and less possible induction of drug resistance. Further or extensive evaluation of these MNPs could provide scaffolds for the development of potent antileishmanial drugs [10]. In the last decade, some of the recent technological advances have necessitated the screening of MNPs against these parasites in an attempt to identify novel antiparasitic agents. Nevertheless, some key knowledge gaps in the MNPs antiparasitic drug discovery pipeline need to be dealt with, such as limited pharmacokinetic and pharmacodynamics studies, and lack of systematic studies (e.g., “time-to-kill” and strains panel assay) [8, 12, 14].

Generally, the problem associated with MNPs discovery has many facets, ranging from ethics and policies associated with samples collection and sample supply, shortcomings of traditional bioassay-guided fractionation approaches, compatibility of some samples to high-throughput screening (HTS) techniques, and the duration, cost, efforts and processes needed before any MNPs can be approved as an effective drug [12].

In the natural environment like marine habitat, apart from culturable microbes, accessing, collecting or recollecting of some samples is a challenge. For instance, when a very potent crude extract is identified from a marine invertebrate such as a sponge, more samples are usually required to get enough crude extract for fractionation and identification of the compound(s). More quantity of the isolated pure compounds is required before they could proceed to other preclinical studies and clinical trials, especially if the chemical synthesis is not yet established or feasible [55]. Then again, factors like sample location and position, season, genotype, differences related to an organism (e.g., age), apparently affect the repeatability or reproducibility, thereby limiting the progression of the identified compounds to the next phase of drug development. However, isolation of marine microbes, heterologous expression of key genes, and semi- or total synthesis of the MNPs, will go a long way in solving some of the issues of sample collection [10].

There is a need to advance sampling techniques to make samples which are not only near-shore like in the deep sea more accessible. Microorganisms from extreme environments, such as high pressure, may require special fermentation technologies to support their cultivation. Microbes only produce what they need, so under normal or artificial conditions, genes responsible for the biosynthesis of many compounds remain silent. These silent or cryptic genes could be activated to increase chemical diversity using strategies like "One strain many compounds” (OSMAC) approach (variation of culture conditions), epigenetic modifications or co-cultivation or metal-stress [56]. For non-culturable and even culturable organisms, advances in genome mining techniques have made it possible to express the genetic information in suitable expression systems without the need to culture the bioactive metabolite producing organism. Additionally, economically feasible synthesis procedure for designing structures with reduced complexity can only be achieved through advances in chemical synthesis and a better understanding of essential structural elements [52].

There are concerns that HTS platforms may not be suitable or compatible with some MNPs, which may be one of the reasons why many MNPs have not proceeded to the next stage. Unlike drug-like synthetic compounds, some crude extracts or fractions are not amenable to screening due to the complex nature of extract. However, this issue of incompatibility could be averted if libraries containing fractions or extracts (with more drug-like properties), or natural product, pure, or semi-synthetic-inspired compounds, are established [8].

For the cytotoxicity screening to be complete using a cell-based technique, it’s necessary to perform other pharmacological tests, by using specific targets as well as by application of high content methods. A very potent drug candidate could be identified if there is a combination of cellular tests with ‘omics’ technologies, which will help to shed light on the mode of action and avoid the failure of the isolated compound later. Researchers should as well be focus initially on checking the toxicology and pharmacokinetic behaviour of the potential compound or drug [18, 57].

MNPs discovery still faces the issue of lack of consensus in bioassay-guided fractionation, especially in terms of fractions collection, isolation and structural elucidation of the bioactive compounds. Nevertheless, some of these challenges may be surmounted with the continuous advancement in chemical analysis techniques such as chromatography (e.g., gas chromatography, thin layer chromatography, high performance layer chromatography), spectrometry (e.g., mass spectrometer) and spectroscopy (e.g., ultraviolet, evaporative light scattering, refractive index, nuclear magnetic resonance), and researchers believe that the techniques could greatly revamp bioactivity guided fractionation, especially for complex extracts [58, 59]. Furthermore, with the evolution of HTS and ‘omics’ techniques (e.g., genomics, proteomics, metabolomics and transcriptomics), computational techniques, and databases for natural products, there should be an increase in the de-replication process, which will foster the identification of novel MNPs and ensuing lead development efforts [58].

## Conclusion

Since it is challenging to treat NTDs and some of the available drugs are becoming obsolete, bioprospecting of marine macro and microorganisms has led to many remarkable milestones especially in the discovery of novel compounds. Due to the complexity of the environment, researchers believe it could be the source of novel compounds with therapeutic potential, for the reason that some of these unique compounds to large extent are produced to ensure their survival in this diverse and often hostile habitat, probably in self-defence. The uniqueness of the environment enables the production of an array of useful metabolites which act as chemical defence as well as display a vast range of bioactivities.

However, despite the broad structural and stereo-chemical diversity of compounds isolated from marine organisms, none of which have entered clinical trials for the management of NTDs and only few undergoing preclinical studies. Few are on preclinical studies. Notably, these reported/isolated compounds with antiparasitic activities still need to undergo extensive biological evaluations and in vivo studies in relevant model systems to ascertain their efficacy, stability and pharmacokinetics as well as safety. The major stumbling block is that large quantities of MNPs are needed for both preclinical studies and clinical trials. Moreover, with the use of semi-synthetic modifications of natural products or the total synthesis of their analogues, synthetic chemists all over the world aim at using these strategies to help overcome the supply issue surrounding MNPs, as well as to enhance their biological properties. Hence, this will help to overcome the supply issue and satisfy the required needs for clinical trials and their eventual development and commercialization.

Around the world, preclinical studies on MNPs continue to identify several novel bioactive marine compounds with therapeutic potentials despite the challenges involved. As more studies on MNPs are being reported, more unique compounds are as well being isolated and identified, and it appears that MNPs armamentarium is becoming sufficient to deliver more potent drugs to the marketplace soon. The currently isolated MNPs with antiparasitic activities need to undergo further biological evaluation, including toxicity, efficacy studies in animal models, pharmacokinetic, as well as structure–activity relationships evaluation.

Finally, regardless of the lack of new classes of drugs against the NTDs that have come to market in recent years, it is evident that exploiting the advantages of recent innovative approaches to drug discovery and establishing constructive collaborations between academia and industry will serve as a foundation for bolstering the drug armamentarium for malaria and neglected tropical diseases.

## Data Availability

Not applicable.
